# Role of Type I Interferon Receptor Signaling on NK Cell Development and Functions

**DOI:** 10.1371/journal.pone.0111302

**Published:** 2014-10-21

**Authors:** Jean Guan, S. M. Shahjahan Miah, Zachary S. Wilson, Timothy K. Erick, Cindy Banh, Laurent Brossay

**Affiliations:** Department of Molecular Microbiology and Immunology and Graduate Program in Pathobiology, Division of Biology and Medicine, Brown University, Providence, Rhode Island, United States of America; Istituto Superiore di Sanità, Italy

## Abstract

Type I interferons (IFN) are unique cytokines transcribed from intronless genes. They have been extensively studied because of their anti-viral functions. The anti-viral effects of type I IFN are mediated in part by natural killer (NK) cells. However, the exact contribution of type I IFN on NK cell development, maturation and activation has been somewhat difficult to assess. In this study, we used a variety of approaches to define the consequences of the lack of type I interferon receptor (IFNAR) signaling on NK cells. Using IFNAR deficient mice, we found that type I IFN affect NK cell development at the pre-pro NK stage. We also found that systemic absence of IFNAR signaling impacts NK cell maturation with a significant increase in the CD27^+^CD11b^+^ double positive (DP) compartment in all organs. However, there is tissue specificity, and only in liver and bone marrow is the maturation defect strictly dependent on cell intrinsic IFNAR signaling. Finally, using adoptive transfer and mixed bone marrow approaches, we also show that cell intrinsic IFNAR signaling is not required for NK cell IFN-γ production in the context of MCMV infection. Taken together, our studies provide novel insights on how type I IFN receptor signaling regulates NK cell development and functions.

## Introduction

Innate lymphoid cells comprise a large number of subsets, including natural killer (NK) cells [1]. NK cells are cytotoxic effector lymphocytes of the innate immune system that develop from the common lymphoid progenitor (CLP) in the bone marrow [2], [3]. The journey from CLP to NK cell starts with the earliest known precursor stage, termed the pre-pro A stage, which are Lin^−^ID2^+^Sca1^+^CD127^+^CD117^low^CD135^−^CD122^−^. Pre-pro B have a similar expression profile, except that they lack CD117 [4], [5]. The relationship between these two precursor populations is currently unclear, but they can both generate efficiently into NK cells *in vitro* and have lost B and T cell potential. After the pre-pro stages, the next step in NK cell development constitutes precursor cells that are Lin^−^CD27^+^CD244^+^CD127^+^CD117^low^CD135^−^CD122^+^, termed rNKP. From here, the rNKP develop into immature NK, and ultimately mature NK cells [6]. A variety of transcription factors that affect NK cell development have been identified and are required at different development stages [7]. Among them, E4bp4 (also known as nfil3) is critical for NK cell production [8]–[10]. During NK cell lineage commitment, NK cell development and function is regulated by activating, inhibitory and cytokine receptor signaling. Among cytokines, type I interferons (IFN; also called IFN-α/β) are expressed rapidly from various cell types following exposure to a variety of infectious agents, and exert critical biological functions, even in the absence of infection [11]. The effects of type I IFN signaling on NK cell development have been difficult to assess due to the pleiotropic nature of these cytokines [12]. In this study, using a variety of approaches, we defined the type I interferon receptor (IFNAR) signaling contribution to NK cell development and function. We found that lack of type I IFN signaling has subtle but noticeable effects on NK cell development. While IFNAR^−/−^ mice have the same number of mature NK cells as B6 mice, the number of NK cell progenitors is significantly decreased in the bone marrow of the deficient animals. We also found that IFNAR^−/−^ mice have a significant increase in the CD27^+^CD11b^+^ NK cell compartment in all organs. However, while this maturation effect is direct in the liver and bone marrow, it is indirect in the spleen and blood, indicating tissue specificity. Finally, using adoptive transfer and mixed bone marrow approaches, we show that NK cell IFN-γ production is not affected by lack of type I IFN signaling in the context of MCMV infection.

## Materials and Methods

### Mice

C57BL/6, B6.SJL, and Rag2-IL2Rγ^−/−^ mice were purchased from Taconic Laboratory Animals and Services, Germantown, NY. IFNAR^−/−^ [13] and littermate control mice backcrossed onto a C57BL/6 background were bred in our animal facility. IFNAR^−/+^ mice were bred, the resultant WT, IFNAR^−/+^ and IFNAR^−/−^ mice were used. All mice were maintained at Brown University in accordance with institutional guidelines for animal care and use.

### Murine Lymphocyte Isolation

Mice were sacrificed by isofluorane treatment. Cardiac puncture was performed prior to the harvesting of the organs. Livers were perfused with PBS-Serum (PBS +1% Fetal Bovine Serum) before harvesting. Spleens were dissociated using a plunger from a 3 mL syringe and lymphocytes were enriched using Lympholyte Cell Separation Media (Accurate Chemical). Livers were dissociated using a gentleMACS Dissociator (Miltenyi Biotec) and lymphocytes were enriched using a 40–70% discontinuous Percoll gradient (GE Healthcare) as previously described [14]. Salivary gland lymphocytes were prepared as described [14]. Briefly, SMGs were removed of all lymph nodes and connective tissue, followed by mincing. Single cell dissociation was performed using one incubation with digestion medium (RPMI 1640 containing 1 mg/ml of collagenase IV (Sigma), 5 mM CaCl2 50 µg of DNase I (Sigma) and 8% FBS) and continuous shaking at room temperature. The digestion mixture was pipetted vigorously to dissociate remaining cells. Supernatant was collected, passed through nylon mesh and the lymphocytes purified by layering on a lympholyte-M gradient. Bone marrow cells were isolated from femur and tibia. Red blood cells were lysed with ammonium chloride lysis buffer.

### Adoptive transfer

Splenic NK cells were isolated from IFNAR^−/−^ (CD45.2^+^) mice and B6.SJL (CD45.1^+^) mice using the STEMCELL NK Cell Isolation Kit, combined in a 1∶1 ratio, and introduced into recipient Rag2-IL2Rγ^−/−^ mice by tail vein injection. The recipient mice were infected with MCMV four hours after transfer, and lymphocytes were isolated from the spleen, liver, and blood 40 hours after infection.

### Mixed bone marrow chimeras

Mixed bone marrow chimeras were prepared as described previously [15]. Briefly, B6.SJL recipient mice were irradiated at 1050 rad. 24 hours later, the irradiated mice were injected with a 1∶1 mixture of DX5 and CD5 depleted bone marrow cells derived from the femurs and tibias of B6.SJL and IFNAR^−/−^ (or littermate heterozygote control) mice. After 7 to 9 weeks, bone marrow chimeras were used for infection and maturation experiments.

### Infection and treatment protocols

Stocks of MCMV clone RVG102 were a gift of Dr. Hamilton [16], Duke University and MCMV Strain # VR1399 was received from American Type Culture Collection (ATCC). MCMV clone RVG102 is recombinant for GFP under the Immediate early gene-1 (ie-1) promoter. Both strains were prepared in the laboratory as previously described from salivary glands [17]. Infections were performed by intraperitoneal injection with 5×10^4^ plaque-forming units (pfu) of MCMV.

### Flow cytometric analysis

Cells were suspended in 1% PBS-serum. Cells were first incubated with the blocking monoclonal antibody (mAb) 2.4G2, and then stained with mAbs specific for cell surface markers for 20 minutes at 4°C. For intracellular staining, cells were fixed with Cytofix/Cytoperm (BD Biosciences) for 20 minutes, and then stained in 1X PermWash (BD Biosciences) for 20 minutes. Events were collected on a FACSAria (BD) or a MacsQuant (Miltenyi Biotec). The resulting data was analyzed using FlowJo software (TreeStar). As a gating strategy, we first gate on lymphocytes based on forward scatter (FSC)-area versus side scatter (SSC)-area. Within this population, doublets are excluded, first by analyzing SSC-height versus SSC-width, then FSC-height versus FSC-width.

### Lymphocyte Counts

Lymphocytes were counted by hemocytometer. Lymphocytes suspended in PBS +1% Serum (GIBCO) were diluted in Trypan Blue (GIBCO). All four quadrants were counted and the averages taken for cell counts. Alternatively, cells were diluted and counted with PI exclusion using the Miltenyi MACSQuant.

### Antibodies and reagents

FITC-DX5, PerCPCy5.5-NK1.1, PE-IFN-γ, APC-CD3, eF450-CD3, eF450-Lin, PE-CD49a, FITC-TCRβ, PE-CD27, PECy7-CD11b, eF780-CD45.2, eF450-CD45.1, FITC-Granzyme-B, APC-Ly49H, FITC-LY49H, PE-TCRβ, APC-CD45.1, eF450-Ki-67, PerCPeF710-CD27, PECy7-NK1.1, eF450-CD11b, AF700-CD3, PECy7-Sca-1, AF700-CD117, AF750-CD127, APC-CD135, FITC-CD244, PE-CD122 were purchased from eBioscience and BioLegend (San Diego, CA).

### Statistical Analysis

Data sets were analyzed for statistical significance using unpaired, two-tailed, Student's t-tests. Values of *p*<0.05 were considered significant. Graphs and statistics were generated and calculated using Prism (GraphPad). Statistical analysis were performed using GraphPad Prism (GraphPad Software, San Diego, CA), where * = p≤0.05, ** = p≤0.005, *** = p≤0.0005, and **** = p≤0.0001.

### Ethics statement

This study was carried out in strict accordance with the recommendations in the Guide for the Care and Use of Laboratory Animals as defined by the National Institutes of Health (PHS Assurance #A3284-01). Animal protocols were reviewed and approved by the Institutional Animal Care and Use Committee (IACUC) of Brown University. All animals were housed in a centralized and AAALAC-accredited research animal facility that is fully staffed with trained husbandry, technical, and veterinary personnel.

## Results

### IFNAR signaling does not affect NK cell number or frequency

**Figure 1:**
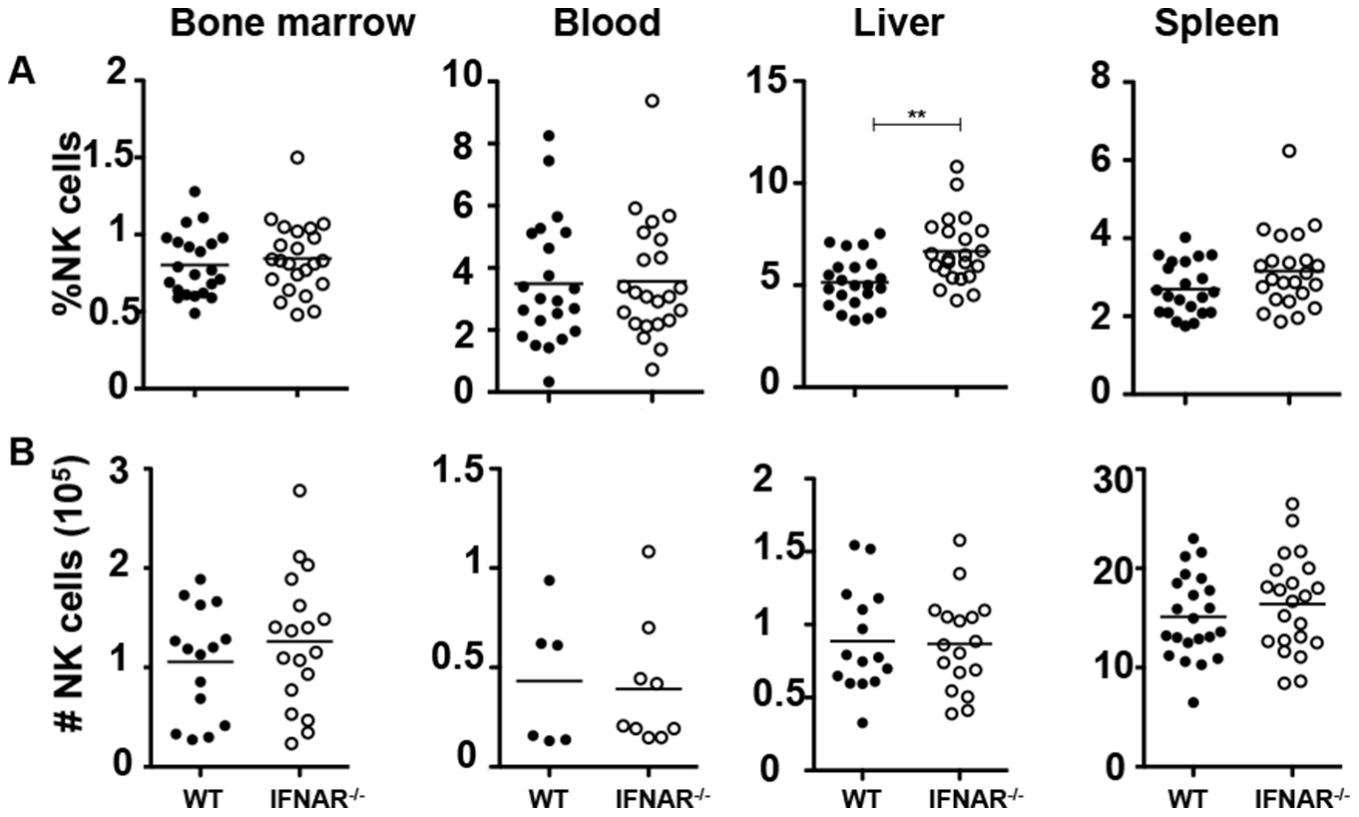
NK cell number and frequency in IFNAR deficient mice. (A) Percentages of the indicated organs containing NK cell (NK1.1^+^CD3^−^ in the lymphocyte gate) populations in the indicated organs from wild-type littermate (black circles), and IFNAR deficient mice (open circles). Data are pooled from at least 5 independent experiments and each dot represents data obtained from one mouse; horizontal lines indicate the mean. (B) Total NK cell numbers of the indicated organs. Data are pooled from at least 5 independent experiments and each dot represents data obtained from one mouse; horizontal lines indicate the mean.

**Figure 2:**
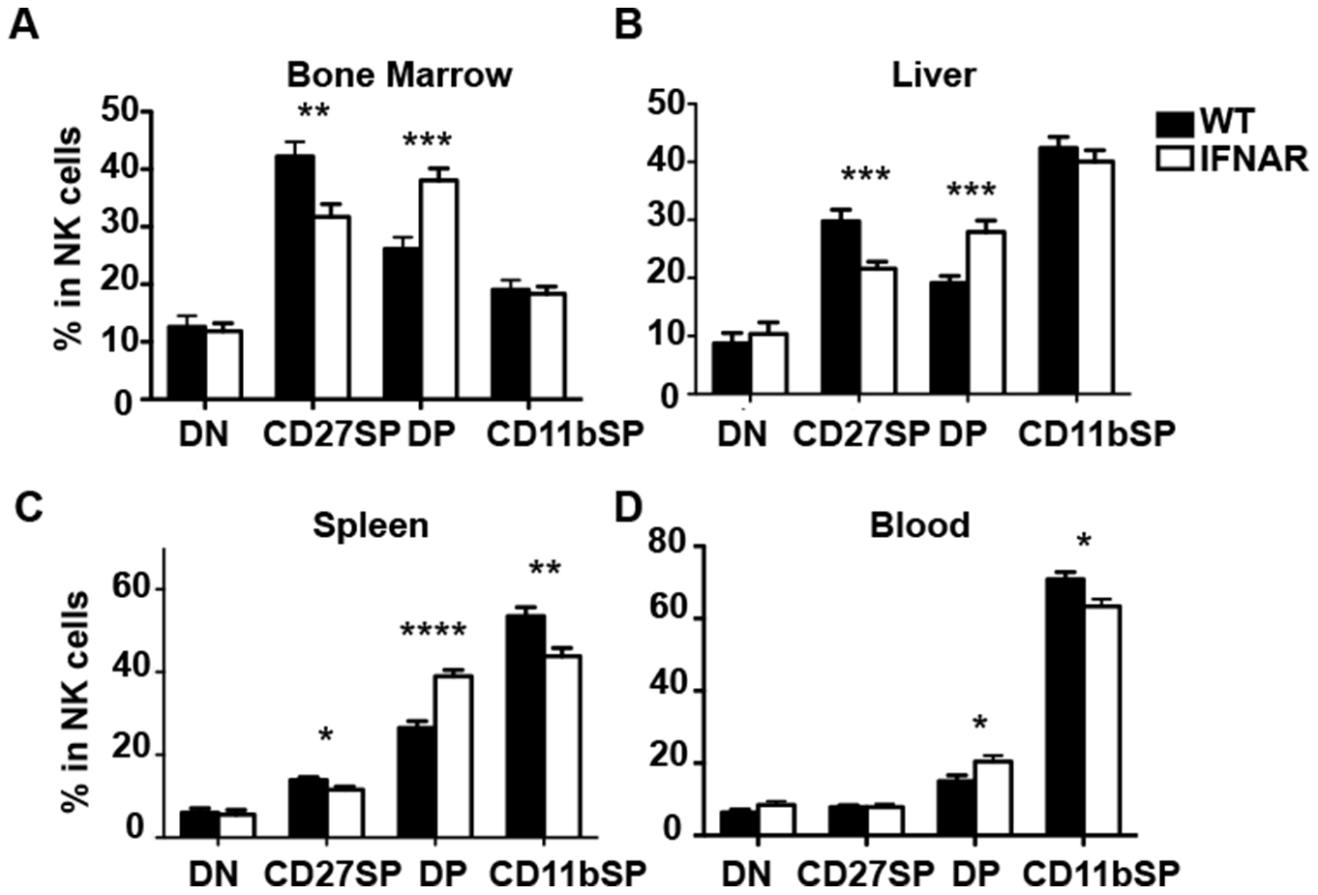
Impaired NK cell development in IFNAR deficient mice. Percent of NK cells (NK1.1^+^CD3^−^) being DN, CD27 SP, DP, and CD11b SP populations in the indicated organs. Representative figures of at least five independent experiments are shown.

We first compared the NK cell cellularity in IFNAR^−/−^ and littermate controls and found there were no significant differences in number or frequency in all tissues tested (Figure 1). Stable distributions of NK cell populations were also found in chimeric mice regardless of the donor bone marrow source, IFNAR^−/−^ or wild-type (Figure S1). We then investigated whether type I interferon receptor signaling had a significant role in the development of bone marrow derived NK cell populations. To examine this, we first looked at the CD11b/CD27 expression of CD3^−^NK1.1^+^ NK cells. These two markers characterize NK cells from least mature to most mature as follows: CD11b^low^CD27^low^, CD11b^low^CD27^high^, CD11b^high^CD27^high^, CD11b^high^CD27^low^ [18]. In IFNAR^−/−^ mice, the double positive CD27^+^CD11b^+^ NK cell population was consistently and significantly increased in the spleen, liver, blood, and bone marrow (Figure 2A–D, and Figure S2). This increase in maturation was also accompanied by a subsequent decrease in the single positive CD27^+^CD11b^−^ NK cell population in all organs, as well as a decrease of the most mature NK cells (CD27^−^CD11b^+^) in the periphery. Thus, although the NK cell populations were unaffected in proportion, IFNAR^−/−^ NK cells are enriched at the CD27^+^CD11b^+^ DP stage. Thus NK cell maturation is affected when IFNAR signaling is abrogated systematically. Notably there is no maturation difference between the wild-type and the *IFNAR^−/+^* (data not shown).

### Pre-pro NK cells are reduced in IFNAR deficient mice

**Figure 3:**
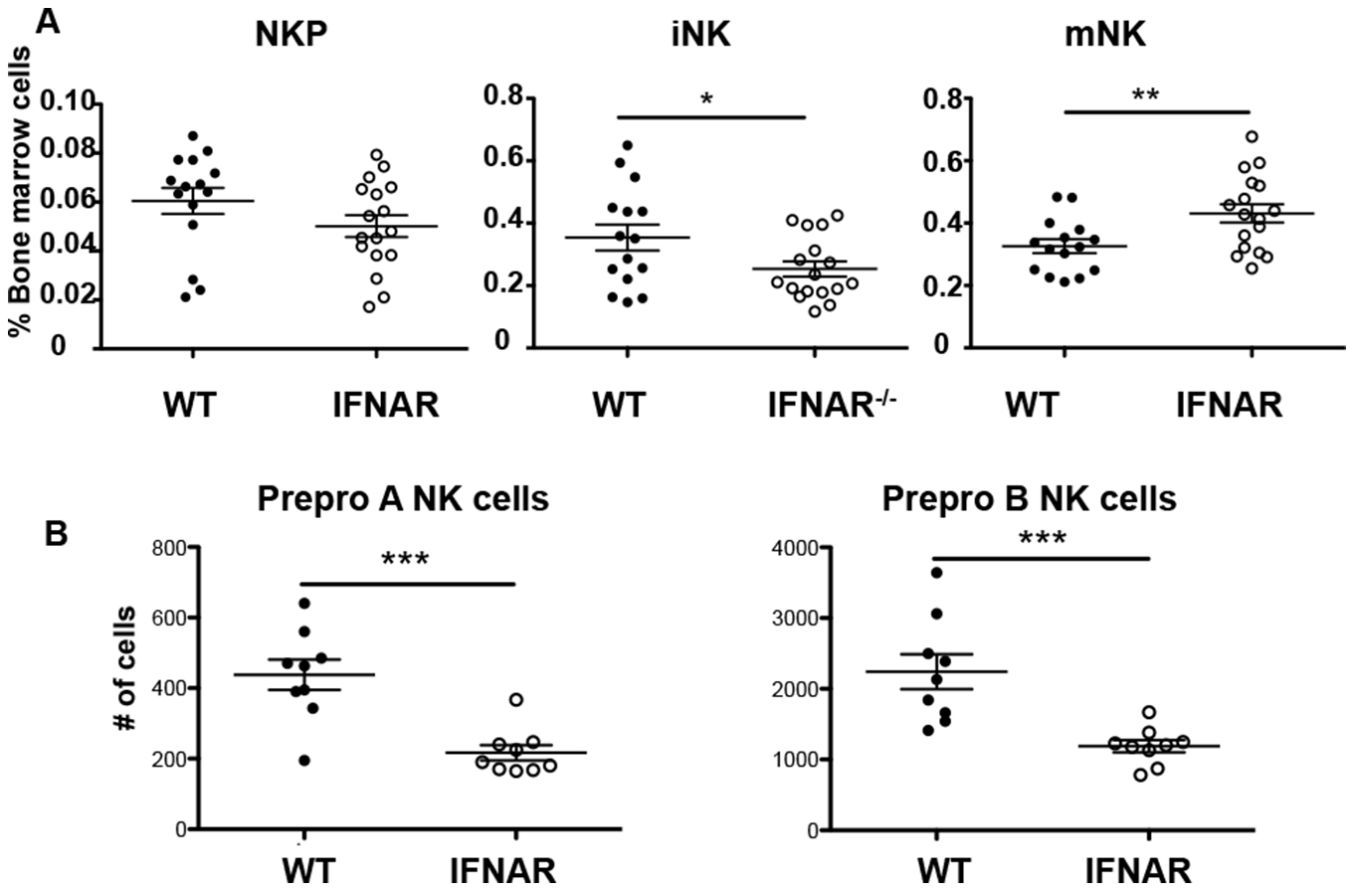
Pre-pro NK cells are reduced in IFNAR deficient mice. (A) Percent NKPs (CD122^+^NK1.1^−^CD3^−^CD11b^−^), iNK cells (CD122^+^NK1.1^+^CD3^−^CD11b^−^), and mNK cells (CD122^+^NK1.1^+^CD3^−^CD11b^+^) among bone marrow lymphocytes derived from littermate wild-type (closed circles), and IFNAR (open circles) mice. Data are pooled from at least 3 independent experiments and each dot represents data obtained from one mouse; horizontal lines indicate the mean. (B) Number of pre-pro NK cells from wild type and IFNAR deficient mice. Data are pooled from at 3 independent experiments and each dot represents data obtained from one mouse.

Using the original definition of NK precursors [19], we found that iNK cells, characterized as CD3^−^CD122^+^NK1.1^+^CD11b^−^, were decreased in the bone marrow of IFNAR^−/−^ mice while mNK cells were increased, shown by the markers CD3^−^CD122^+^NK1.1^+^CD11b^+^ (Figure 3). The increase of mNK in the bone marrow is due to the accumulation of CD11b^+^CD27^+^ NK cells, also seen in the periphery. Interestingly, using the recently defined characterization of pre NK cell progenitors [4], [5] we found that the number of prepro NK A and B were both significantly decreased in the IFNAR^−/−^ mice (Figure 3). This is consistent with previous findings in hematopoietic stem cells (HSCs) showing that type I IFN induce proliferation of HSCs [20], [21].

### Tissue resident NK cells are not affected by the absence of IFNAR

**Figure 4:**
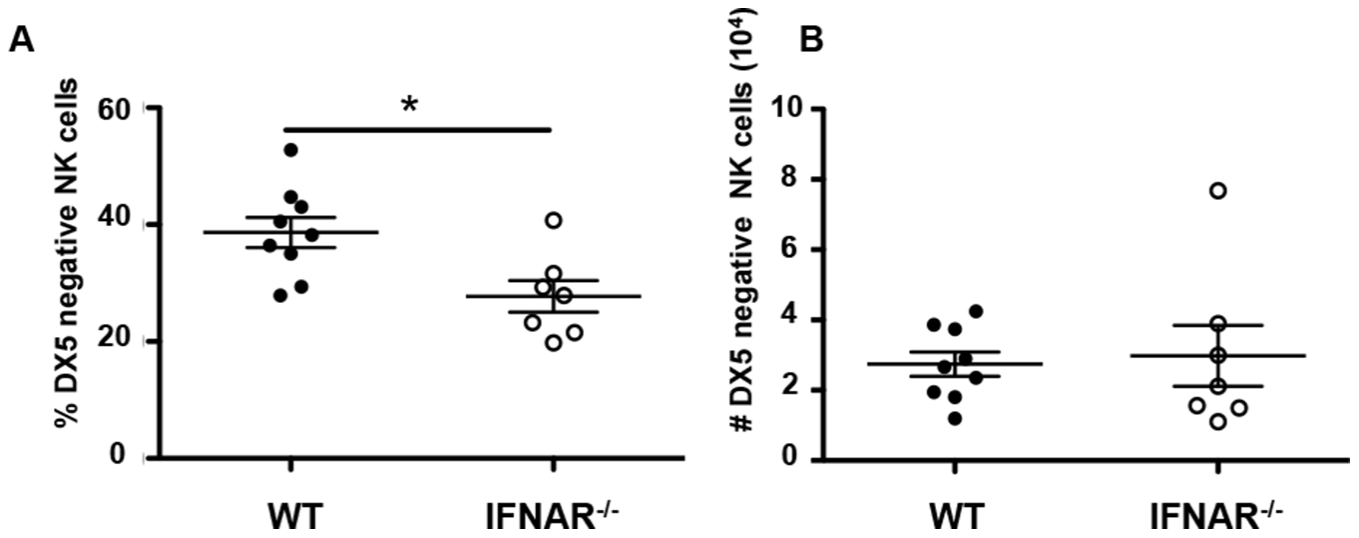
Liver resident NK cells in IFNAR deficient mice. Percentages (A) and number (B) of the liver resident NK cell (NK1.1^+^CD3^−^DX5^−^ in the lymphocyte gate) populations from wild-type littermate (closed circles), and IFNAR deficient mice (open circles). Data are pooled from at least 3 independent experiments and each dot represents data obtained from one mouse.

Tissue NK cells, which include liver resident NK cells, salivary gland NK cells, skin NK cells, and uterus NK cells are beginning to be characterized [22]. We first analyzed the newly characterized liver resident NK cells [23]–[25]. Although the frequency of liver DX5-negative NK cells was significantly decreased in IFNAR deficient mice, we found that the number of liver IFNAR^−/−^ DX5 negative NK cells was similar to wild-type DX5 negative NK cells (Figure 4). We also characterized salivary glands NK cells [14] in IFNAR deficient mice and found no significant difference in frequency between the wild-type and the IFNAR^−/−^ animals (data not shown). Thus, type I interferon appears to be dispensable for the development of tissue resident NK cells.

### IFNAR signaling affects NK cell maturation intrinsically in the bone marrow and liver

**Figure 5:**
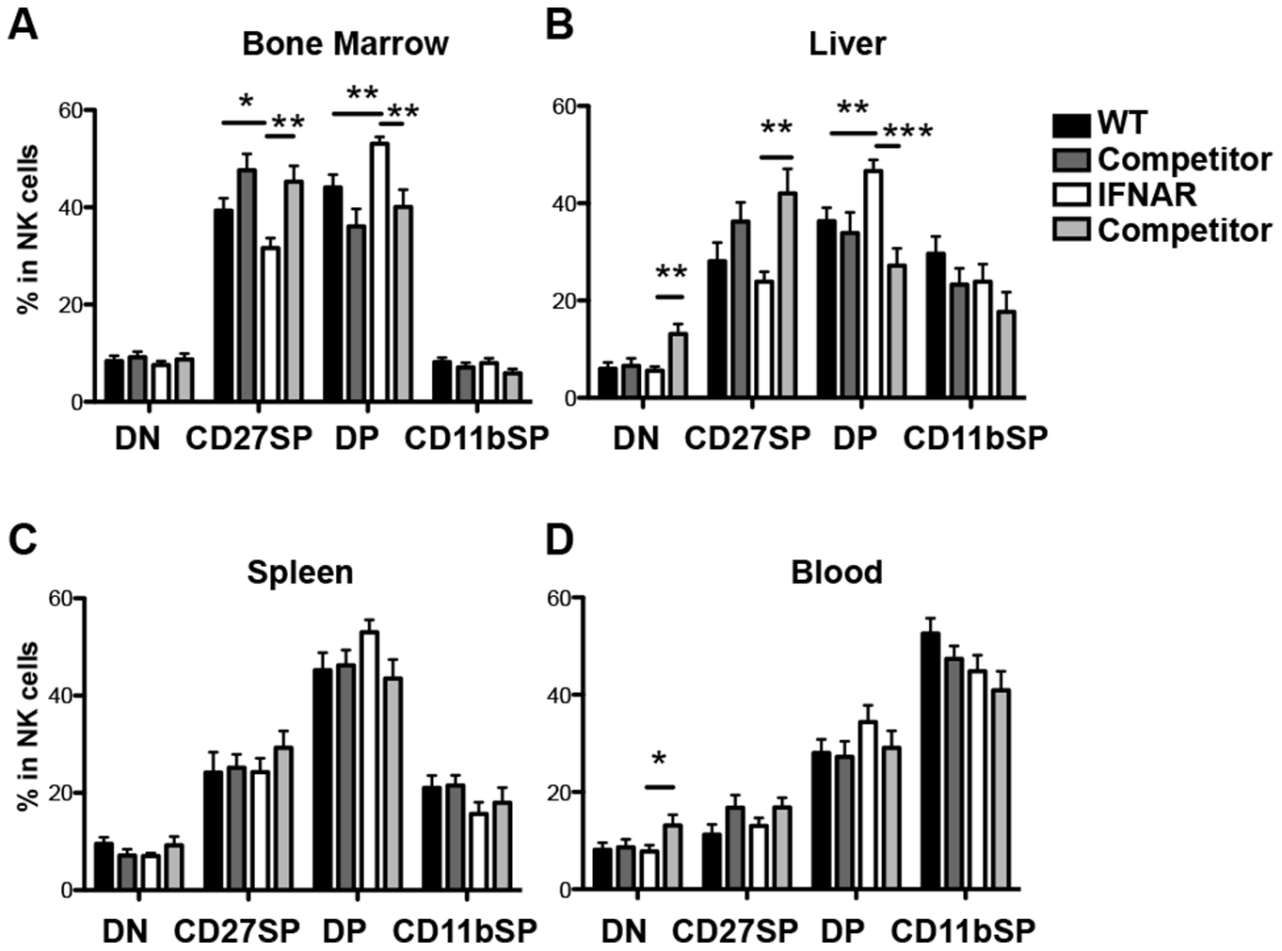
IFNAR signaling regulates NK cell maturation intrinsically in liver and the bone marrow. Percent of NK cells (NK1.1^+^CD3^−^ in the lymphocyte gate) expressing DN (CD27^−^CD11b^−^), CD27 SP (CD11b^−^CD27^+^), DP (CD27^+^CD11b^+^), and CD11b SP (CD27^−^CD11b^+^) populations in the indicated organs derived from mixed bone marrow chimeras. Data are pooled from at least 5 independent experiments. Error bars indicate SEM.

To determine the exact contribution of IFNAR signaling on NK cell development and maturation, we generated a series of mixed bone marrow chimeras. IFNAR^−/−^ (or IFNAR^+/−^) bone marrow and wild-type competitor bone marrow were transferred at a 1∶1 ratio into lethally irradiated wild-type recipients. The phenotype observed in the straight IFNAR deficient mice was conserved in the bone marrow and liver of the chimeric mice with a decrease of CD27^+^ NK cells and an accumulation of the DP CD11b^+^/CD27^+^ NK cells (Figure 5). A representative staining is shown in Figure S3. This phenotype was seen when we compared the IFNAR^−/−^ NK cells and their wild type competitor within the same mice as well when we compared the NK cells from WT:WT chimeric mice to the IFNAR^−/−^ NK cells from the WT:IFNAR^−/−^ chimeras. In contrast in the blood and spleen, no differences in the CD27^+^SP, CD11b^+^CD27^+^DP, and CD11b^+^SP maturation stages were found (Figure 5). These data suggest that IFNAR signaling affects NK cell maturation intrinsically in the bone marrow and liver, but is compensated by other mechanisms in the periphery.

### IFNAR^−/−^ NK cells remain open to IFN-γ production during viral infection

**Figure 6:**
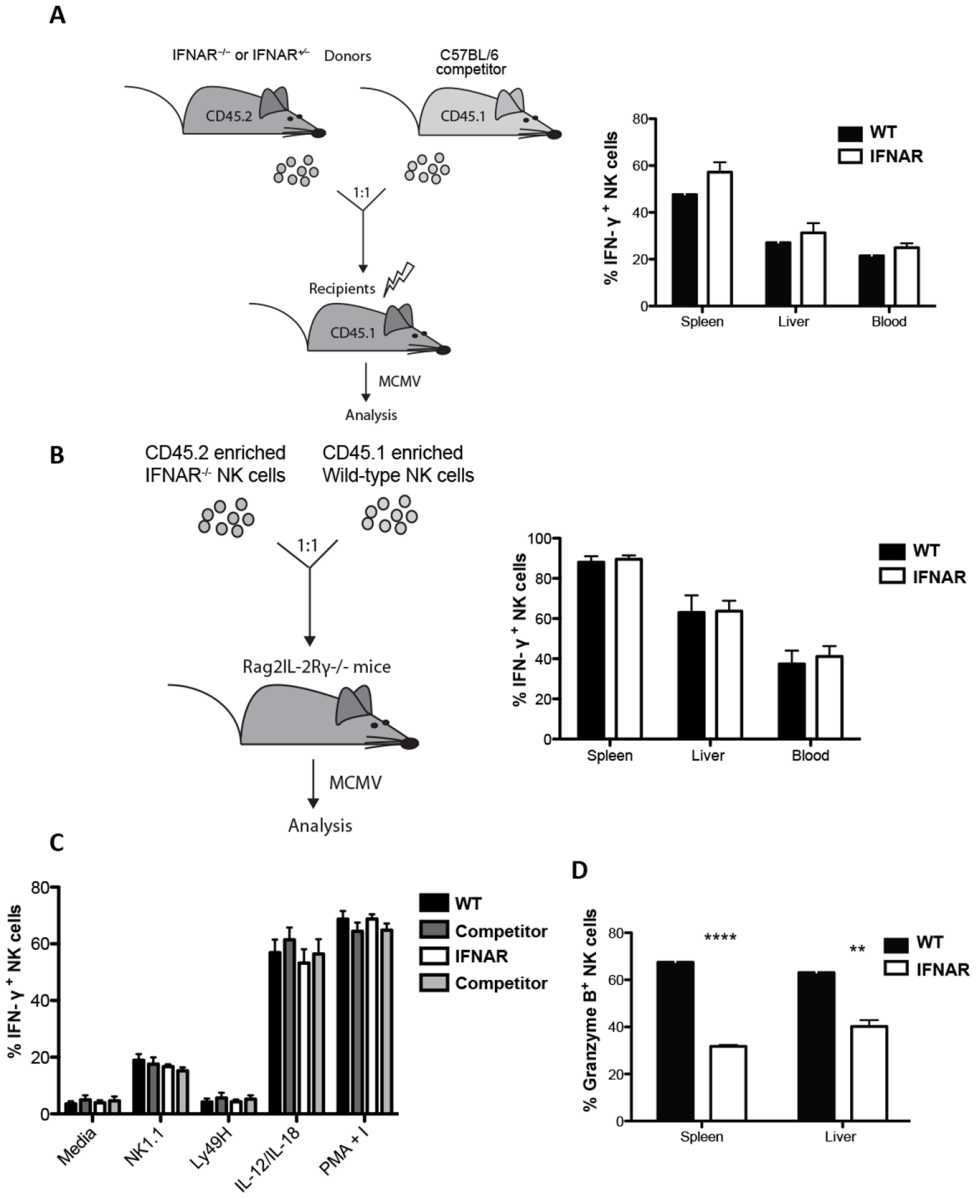
IFNAR signaling is not intrinsically required for NK cell IFN-γ production during MCMV infection. (A) Intracellular IFN-γ in NK cell populations (NK1.1^+^CD3^−^ in the lymphocyte gate) from mixed bone marrow chimeric mice infected with MCMV. Representative of 4 independent experiments with 3 mice per group. (B) Intracellular IFN-γ in adoptively transferred NK cell populations (NK1.1^+^CD3^−^ in the lymphocyte gate) from Rag2IL-2Rγ^−/−^ mice infected with MCMV. Representative of 2 independent experiments with 3 mice per group. (C) Intracellular IFN-γ in NK cell populations (NK1.1^+^CD3^−^ in the lymphocyte gate) from total splenocytes incubated with antibody-coated plates using anti-Ly49H, and anti-NK1.1, or with a mix of IL-12/IL-18 and PMA and ionomycin (P/I) for six hours are assessed after gating on donor cells. Representative of 5 independent experiments with 3 mice per group. (D) Intracellular Granzyme B in NK cell populations (NK1.1^+^CD3^−^ in the lymphocyte gate) from mixed bone marrow chimeric mice infected with MCMV. Representative of 2 independent experiments with 3 mice per group.

Type I IFN exert pleiotropic effects on different cell subsets, making it difficult to assess the effect of these cytokines on NK cell functional responses. To circumvent this, we have designed two approaches. First, we used mixed bone marrow chimeras (Figure 6A), which allowed us to compare the response of wild-type NK cells and IFNAR^−/−^ NK cells to viral infection in the same environment. IFNAR^−/−^ mice are extremely sensitive to MCMV infection and succumb at a non-lethal dose, even in the resistant B6 background (data not shown and [26]). However, mixed bone marrow chimeras (WT:IFNAR^−/−^) in B6 background are less sensitive to MCMV infection (data not shown). We therefore infected chimeric mice with MCMV and compared the responses of wild type NK cells and IFNAR^−/−^ NK cells, which have developed in the same environment. Both populations of NK cells produced IFN-γ at the same level (number of cells producing IFN-γ and amount produced per cell) regardless of the expression of the IFNAR at their cell surface (Figure 6A). As a second approach, we adoptively transferred equal numbers of enriched wild type NK cells and IFNAR^−/−^ NK cells into Rag2IL-2Rγ^−/−^ mice, which lack B, T, and NK cells, and simultaneously infected the recipient mice with MCMV (Figure 6B). Similarly to the chimeric mice, we found no significant difference in IFN-γ production by adoptively transferred IFNAR^−/−^ and wild type NK cells (Figure 6 B). Finally, IFN-γ release by *ex vivo* splenic NK cells from mixed bone marrow chimeric mice upon crosslinking with NK1.1 and Ly49H was similar in wild type NK cells and IFNAR^−/−^ NK cells (Figure 6C). This was also the case during activation by IL12/IL18 or PMA treatment (Figure 6C). Altogether, our data demonstrate that IFNAR signaling is not required intrinsically for NK cell IFN-γ response during MCMV infection. However, as reported by others [27], IFNAR^−/−^ NK cells produce significantly less granzyme B than wild-type competitor NK cells in MCMV infected chimeric mice (Figure 6D).

## Discussion

In this study, we have examined the role of IFNAR signaling during NK cell development and maturation, which has been somewhat difficult to delineate due to the pleiotropic nature of these cytokines [11]. Using the recently defined characterization of NK cell progenitors [4], [5], we unexpectedly found that IFNAR deficient mice have a lower number of progenitor NK cells than their wild-type counterparts. In the absence of infection, type I IFN are produced constitutively at low levels by a variety of cell types. The decreased number of NK cell progenitors in IFNAR deficient mice is in agreement with recent studies showing that type I IFN induce proliferation in hematopoietic stem cells [20], [21]. While it is well known that type I IFN induces the expression of IL-15 [28], [29], the effect observed here cannot be attributed to a lack of response to IL-15, since pre-pro NK cells do not express CD122. Once committed to the NK lineage, we found that NK cell number in the bone marrow from IFNAR deficient mice is comparable to wild type animals, suggesting that redundancy with other stimuli occurs at this stage.

Our results also demonstrate that the requirement of type I interferon receptor signaling at different stages of NK cell development is tissue specific. We found that the effect of type I interferon on NK cell maturation is indirect in the blood and spleen but direct in the liver and bone marrow. In fact, in the periphery, wild-type and IFNAR^−/−^ NK cells appear to be developmentally indistinguishable, suggesting that other signals have compensated for the lack of IFNAR on NK cells. In a recent study using conditional NCR-1 cre-IFNAR^−/−^ mice, no NK cell maturation defect was found [30]. However, NKp46 is expressed relatively late in NK cell differentiation [6], possibly at a time when the effects of IFNAR signaling on NK cell maturation have already occurred. In bone marrow and liver, it is unclear how the double positive CD27^+^CD11b^+^ NK cells fill the niche left open from the lack of type I interferon intrinsically regulated NK cells. In these organs it is possible that the significant increase in the double positive CD27^+^CD11b^+^ NK cell population compensates for the lack of interferon signaling, as double positive NK cells have been shown to be more sensitive to cytokine stimulation and proliferation [31]. Therefore, it appears to be a buffering mechanism for NK cell development to retain a stable population of NK cells in different degrees of type I interferon signaling. This potentially indicates that evolutionarily favorable feedback mechanisms may be in place to buffer type I IFN signaling for NK cell populations, which is greatly increased in viral infection and in some cases chronically dysregulated.

The identification of new innate lymphoid cell (ILC) subsets has been exponential in recent years. Although the classification of NK cells as a member of ILC1 is still a matter of debate [22], it appears there are alternative developmental pathways for NK cells at least in liver and bone marrow [24]. Our results suggest that interferon receptor signaling is dispensable for the development of the recently described tissue resident NK cells, as well as salivary gland NK cells.

The direct role of IFNAR signaling during viral infection has been somewhat controversial. While one study described a weak direct NK cell response to type I IFN during MCMV infection [32], others have shown a critical and direct type I IFN role during Vaccinia virus [33], [34] or HSV infections [35]. Notably, using IFNAR^−/−^ mice in the 129 background and DX5 to identify NK cells, NK cell IFN-γ was not affected *in vitro* [36]. To eliminate any indirect extrinsic effects of type I IFN, we used two different approaches that allowed us to compare the response of wild type and IFNAR deficient NK cells in the same environment. As we failed to show a significant difference in NK cell IFN-γ production following infection, our data argue that a direct type I IFN effect on NK cells is weak or subtle, at least during MCMV infection. Therefore, the impaired NK cell response to viral infection when IFNAR is systematically deleted is indirect and likely due to dendritic cell priming as previously reported [27], [32]. However, and in agreement with others [27], in the absence of IFNAR signaling, NK cell granzyme B production is decreased suggesting that NK cell granzyme B production is dependent in part of IFNAR signaling, perhaps due to impaired granzyme accumulation [37]. To summarize, we conclude that absence of type I IFN signaling affects the development of NK cell progenitors, the maturation of NK cells in a tissue specific manner, and the development of tissue resident NK cells. However, we found that type I IFN has no direct effect on IFN-γ production by NK cells during viral infection.

## Supporting Information

Figure S1 **NK cell number and frequency in chimeric mice.** Percent (A) and total NK cells (B) (NK1.1^+^CD3^−^ in the lymphocyte gate) present in the indicated organs from the different chimeric mice. Data are pooled from at least 3 independent experiments and each symbol indicates an individual mouse; horizontal lines indicate the mean.
(TIF)

Figure S2 **Altered NK cell maturation in IFNAR^−/−^ mice.** Flow cytometry of CD27 vs CD11b expression in NK cells of the indicated organs from IFNAR^−/−^ and littermate wild-type control mice. Data are representative of at least 5 experiments.
(TIF)

Figure S3 **NK cell maturation is affected intrinsically in liver and the bone marrow.** (A) Gating strategy to separate NK cells from IFNAR^−/−^ and wild type competitor donors. (B) Flow cytometry of CD27 vs CD11b expression in NK cells of the indicated organs. Data are representative of at least 5 experiments.
(TIF)
