# Supplementary figures and images for: Role of Type I Interferon Receptor Signaling on NK Cell Development and Functions
Source: PLoS One. 2014 Oct 21;9(10):e111302. doi: 10.1371/journal.pone.0111302 (PMC4205023; doi:10.1371/journal.pone.0111302)

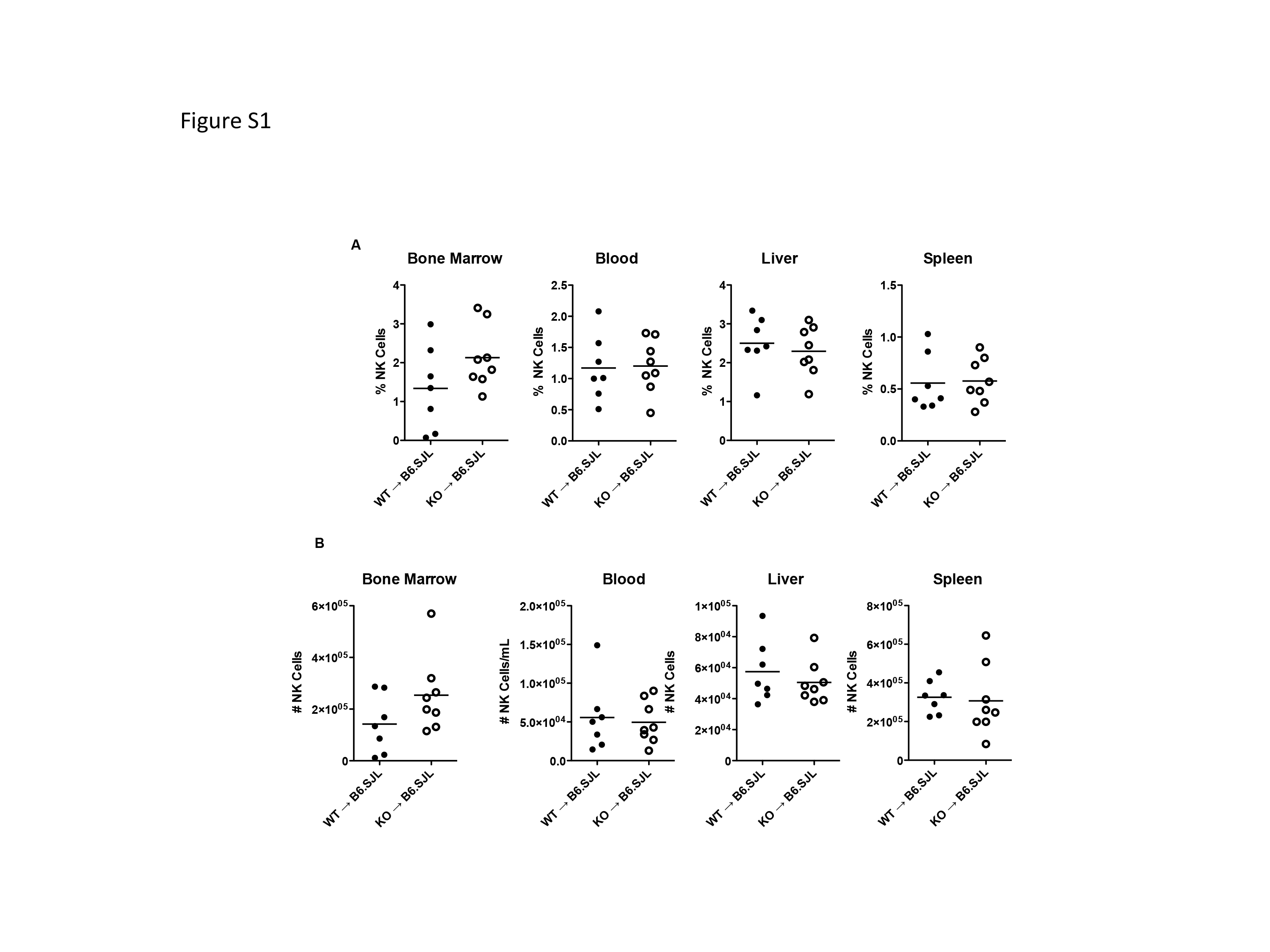

Supplement: Figure S1 — NK cell number and frequency in chimeric mice. Percent (A) and total NK cells (B) (NK1.1+CD3− in the lymphocyte gate) present in the indicated organs from the different chimeric mice. Data are pooled from at least 3 independent experiments and each symbol indicates an individual mouse; horizontal lines indicate the mean. (TIF) [file pone.0111302.s001.tif]

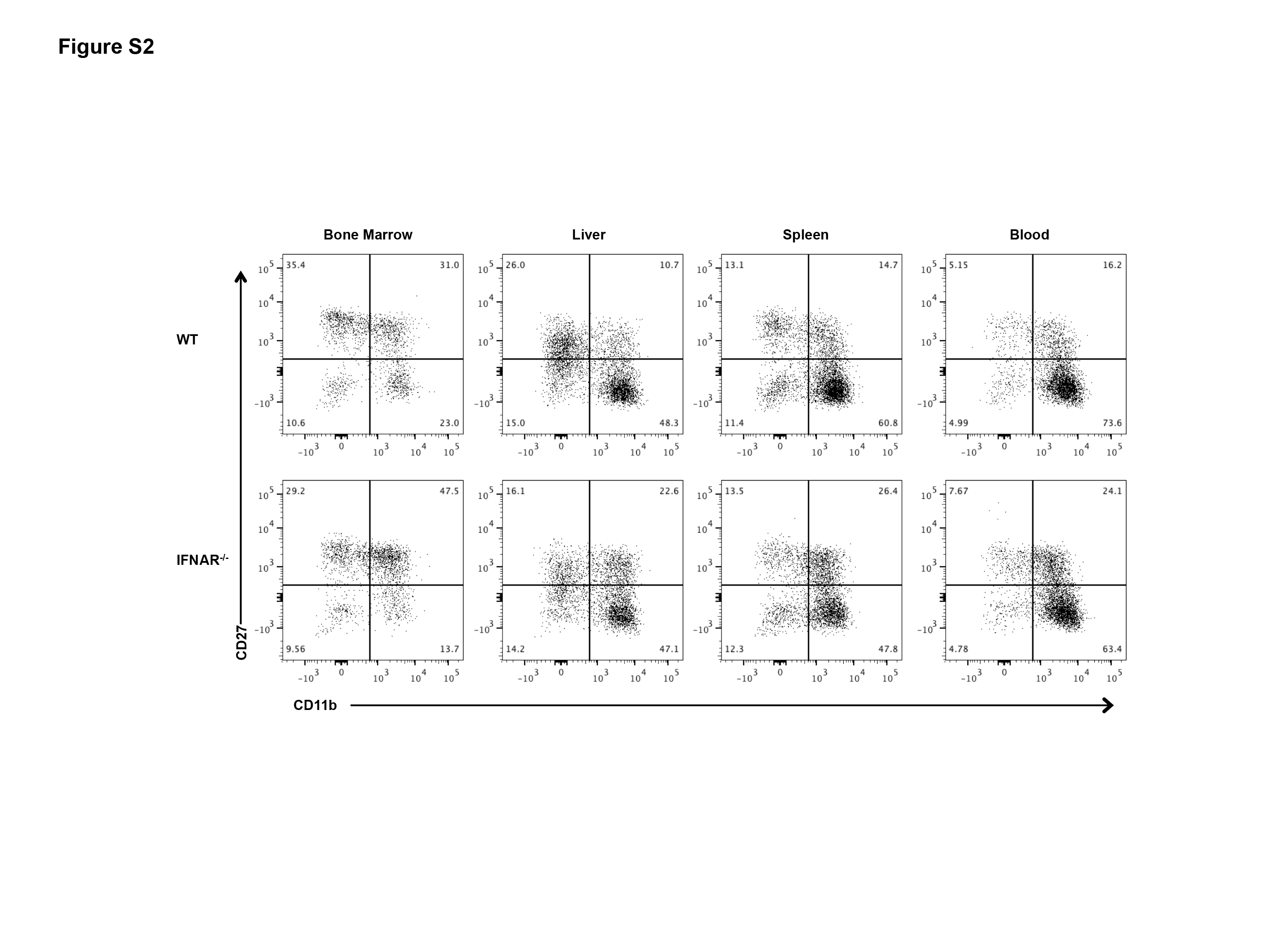

Supplement: Figure S2 — Altered NK cell maturation in IFNAR−/− mice. Flow cytometry of CD27 vs CD11b expression in NK cells of the indicated organs from IFNAR−/− and littermate wild-type control mice. Data are representative of at least 5 experiments. (TIF) [file pone.0111302.s002.tif]

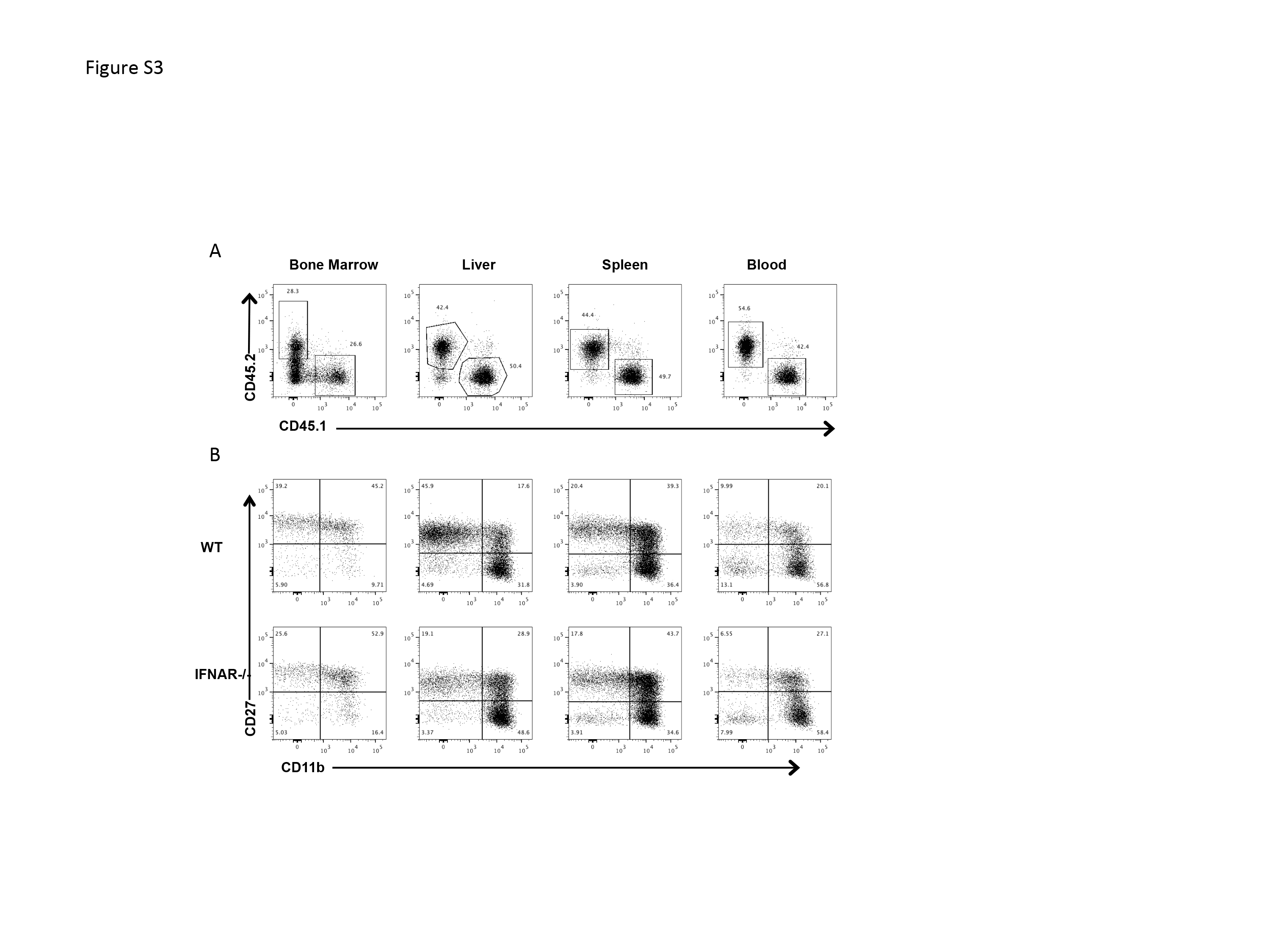

Supplement: Figure S3 — NK cell maturation is affected intrinsically in liver and the bone marrow. (A) Gating strategy to separate NK cells from IFNAR−/− and wild type competitor donors. (B) Flow cytometry of CD27 vs CD11b expression in NK cells of the indicated organs. Data are representative of at least 5 experiments. (TIF) [file pone.0111302.s003.tif]
